# Dilemmas in generic delimitation of *Senegalia* and allies (Caesalpinioideae, mimosoid clade): how to reconcile phylogenomic evidence with morphology and taxonomy?

**DOI:** 10.3897/phytokeys.205.79378

**Published:** 2022-08-22

**Authors:** Vanessa Terra, Jens J. Ringelberg, Bruce Maslin, Erik J. M. Koenen, John Ebinger, David Seigler, Colin E. Hughes

**Affiliations:** 1 Instituto de Ciências Agrárias, Universidade Federal de Uberlândia, Rodovia LMG 746, Km01, s/n, Bloco 1A, sala 413, 38500-000, Monte Carmelo, Minas Gerais, Brazil Universidade Federal de Uberlândia Monte Carmelo Brazil; 2 Department of Systematic and Evolutionary Botany, University of Zurich, Zollikerstrasse 107, CH-8008, Zurich, Switzerland University of Zurich Zurich Switzerland; 3 Western Australian Herbarium, Department of Biodiversity, Conservation and Attractions, Bentley Delivery Centre, PO Box 104, WA, 6983, Australia Western Australian Herbarium Bentley Delivery Centre Australia; 4 Emeritus Professor of Botany, Eastern Illinois University, Charleston, IL 61920, USA Université Libre de Bruxelles Bruxelles Belgium; 5 Department of Plant Biology, University of Illinois, Urbana, Illinois 61801, USA Eastern Illinois University Charleston United States of America; 6 Present address: Evolutionary Biology & Ecology, Université Libre de Bruxelles, Faculté des Sciences, Campus du Solbosch - CP 160/12, Avenue F.D. Roosevelt, 50, 1050 Bruxelles, Belgium University of Illinois Urbana United States of America

**Keywords:** Cytonuclear discordance, Fabaceae, Leguminosae, *Mariosousa*, Mimosoideae, *Parasenegalia*, *Pseudosenegalia*

## Abstract

*Senegalia* comprises 219 species distributed in tropical and subtropical regions of North and South America, Africa, Asia and Australia. Two sections are currently recognised within *Senegalia* and these are most readily distinguished by the differences in disposition of their cauline prickles, i.e. sect. Senegalia with prickles at or near leaf nodes and sect. Monacanthea with mostly internodal prickles. Previous phylogenetic studies, based primarily on small numbers of plastid DNA loci, found *Senegalia* to be monophyletic with two large subclades corresponding to the sections. Here, we present new phylogenomic evidence from 997 single-copy nuclear gene sequences for a small, but representative set of species. These new analyses show that *Senegalia* is non-monophyletic, but instead, forms a grade that is paraphyletic with respect to the remainder of the ingoid clade (i.e. Ingeae + *Acacia* s.s. + *Acaciella*), comprising two well-supported subclades most likely representing the same clades as found in previous phylogenetic studies of the genus and, interspersed between these, a third, moderately supported clade, comprising the genera *Mariosousa*, *Pseudosenegalia* and *Parasenegalia*. In marked contrast to the nuclear phylogeny, the two *Senegalia* clades are sister groups in the plastid phylogeny, based on analyses of 72 chloroplast genes, rendering the genus monophyletic, based on plastid data alone. We discuss this new evidence that *Senegalia* is non-monophyletic in relation to the marked cytonuclear discordance, high gene tree conflict and lack of resolution across this senegalioid grade and review the consistency of the key morphological characters distinguishing the two sections of *Senegalia*. We conclude that it is likely that *Senegalia* will need to be split into two (or possibly more) genera: a re-circumscribed *Senegalia* s.s. that corresponds to the existing Senegaliasect.Senegalia plus the *S.ataxacantha* group (Senegaliasect.Monacanthea s.s.; future studies may show that this group warrants generic status) and a new genus corresponding to the remainder of sect. Monacanthea (here designated as Senegaliasect.Monacanthea p.p.). However, re-delimiting *Senegalia* now would be premature given that the key morphological characters are not fully congruent with the two sections and pending denser phylogenetic sampling of taxa. A judiciously selected list of critical taxa is presented to facilitate future phylogenomic studies. Finally, we discuss the identity of *Albizialeonardii*, which is also placed in this senegalioid grade in these new phylogenomic analyses and place it in synonymy with *Parasenegaliavogeliana*.

## Introduction

*Senegalia* Raf. was segregated from *Acacia* Mill. by Rafinesque (1838) with a very brief description. However, the genus was subsequently overlooked or ignored for almost 100 years, until it was resurrected by Britton and Rose (1928), alongside the newly-segregated genus *Acaciella* Britton & Rose, in their treatment of *Acacia* for the *Flora of North America*. However, despite this recognition by Britton and Rose (1928), *Senegalia* was subsequently ignored (Pedley 1987) and Bentham’s (1875) delimitation of *Acacia* sensu lato (s.l.) as a broadly circumscribed pantropical genus persisted until the reclassification of *Acacia* by Pedley (1986).

Pedley (1986) divided *Acacia* into three genera: *Acacia* sensu stricto (s.s.), *Senegalia* and *Racosperma* Mart. These genera corresponded to the three subgenera of *Acacia* s.l. recognised by Vassal (1972), namely subg. Acacia (now *Vachellia* Wight & Arn.), subg. Aculeiferum Vassal (now *Senegalia* and allied genera) and subg. Phyllodineae DC. (syn. subg. Heterophyllum Vassal and *Racosperma*, now *Acacia* s.s.), respectively. Although Pedley’s classification was not immediately adopted, over the following two decades, a series of molecular and morphological phylogenetic analyses demonstrated that *Acacia* s.l. was polyphyletic and could not be sustained as a single genus (e.g. Luckow et al. 2003; Miller and Seigler 2012). While these analyses confirmed the monophyly of *Vachellia* and Acacia s.s., Vassal’s subg. Aculeiferum formed a paraphyletic grade. Thus, while *Senegalia* was again resurrected, its delimitation remained problematic. Subsequently, more densely sampled phylogenetic analyses of molecular and morphological data led to the segregation (or resurrection) of four small New World genera, namely *Acaciella* (Rico-Arce and Bachman 2006), *Mariosousa* Seigler & Ebinger (Seigler et al. 2006), *Parasenegalia* Seigler & Ebinger (Seigler et al. 2017) and *Pseudosenegalia* Seigler & Ebinger (Seigler et al. 2017). These four genera all differ from *Senegalia* in lacking cauline and foliar prickles (Miller et al. 2017). Recent phylogenomic analyses (Koenen et al. 2020; Ringelberg et al. 2022) have shown that *Senegalia*, *Mariosousa*, *Parasenegalia* and *Pseudosenegalia* together form a poorly-resolved paraphyletic grade (see below), while *Acaciella* is placed in the *Calliandra* clade sensu Koenen et al. (2020) where it is sister to a clade comprising *Calliandra* Benth. and *Afrocalliandra* E.R. Souza & L.P. Queiroz and is not discussed further in this paper.

**Figure 1.**
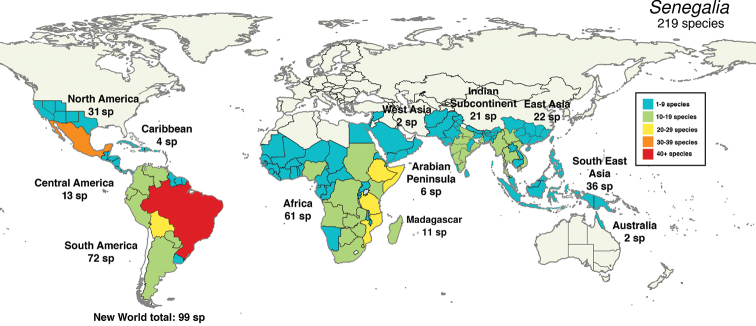
Global distribution of *Senegalia*. Species numbers derived from Maslin and Wilson (2021) at WorldWideWattle website (http://worldwidewattle.com).

*Senegalia* today comprises 219 species (235 taxa) distributed pantropically (Fig. 1) with 99 species in the Americas, 68 species in Africa plus Madagascar, 57 species in Asia [i.e. Arabian Peninsula to East and Southeast Asia (including Papua New Guinea)] and two species in Australia and with particular hotspots of species richness in Brazil (63 species), Mexico (30 species), East Asia (China, 22 species) and east Africa (e.g. Somalia, 21 species; Mozambique, 20 species) (Fig. 1).

Two sections are currently recognised within *Senegalia* (fide Maslin et al. 2019), sect. Senegalia (armed with cauline prickles at or near leaf nodes) and sect. Monacanthea (Vassal) Maslin (prickles mostly internodal). The monophyly of *Senegalia* was supported by recent phylogenetic analyses of plastid DNA sequences (Bouchenak-Khelladi et al. 2010; Kyalangalilwa et al. 2013; Boatwright et al. 2015), consistently recovering two well-supported clades that are sister to each other. These two clades were also recovered in an analysis of plastid loci combined with nrDNA ITS sequences by Terra et al. (2017). A more recent phylogenomic study using genome-scale nuclear sequence data (Koenen et al. 2020) also robustly supported the same two clades, but revealed that these are not sister to each other, rejecting the monophyly of *Senegalia*.

## New phylogenomic evidence

**Figure 2.**
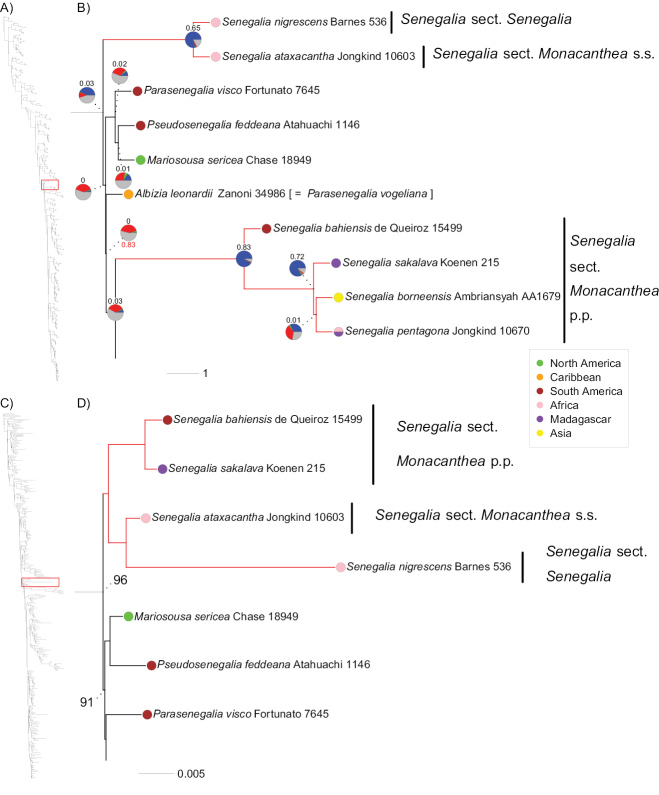
Cytonuclear discordance in *Senegalia* and allies **A** phylogeny of Caesalpinioideae, showing the placement of *Senegalia* and closely related genera (boxed in red) within the subfamily (Ringelberg et al. 2022). The phylogeny contains 420 taxa (excluding outgroups) and is based on 997 nuclear genes analysed using ASTRAL (Mirarab 2019) **B** phylogeny of *Senegalia* and allies (details from **A**). Pie charts at nodes show the fraction of gene trees supporting that bipartition in blue, the fraction of gene trees supporting the most likely alternative configuration in green, the fraction of gene trees supporting additional conflicting configurations in red and the fraction of uninformative gene trees in grey. Numbers above pie charts show quartet-based Extended Quadripartition Internode Certainty scores (Zhou et al. 2020), numbers below pie charts the outcome of ASTRAL’s polytomy test, which tests the null hypothesis that a branch should be replaced by a polytomy (only values > 0.05 are shown). In both **A** and **B**, branch lengths are expressed in coalescent units and terminal branches were assigned an arbitrary uniform length for visual clarity **C** plastid gene tree of Caesalpinioideae showing the placement of *Senegalia* and closely-related genera (boxed in red) within the subfamily (Ringelberg et al. 2022). The phylogeny contains 381 taxa (excluding outgroups) and is based on 72 concatenated plastid genes analysed using RAxML (Stamatakis 2014) **D** plastid phylogeny of *Senegalia* and allies (details from **C**). Numbers at nodes show bootstrap support values for nodes that are not fully supported. In both **C** and **D**, branch lengths reflect nucleotide substitutions. In both **B** and **D**, the root is not drawn to scale.

In this paper, we review new phylogenomic evidence derived from analyses of sequences of 997 nuclear and 72 plastid genes for 422 taxa of subfamily Caesalpinioideae that sampled all but one of the 90 genera in the mimosoid clade (Ringelberg et al. 2022). These analyses are based on a slightly modified set of the same *Mimobaits* genes used by Koenen et al. (2020), but sampled four more species of *Senegalia*, plus representatives of *Mariosousa*, *Parasenegalia* and *Pseudosenegalia* (Fig. 2) which were not included by Koenen et al. (2020). Taxon sampling across the nuclear and plastid phylogenies of Ringelberg et al. (2022) is not identical (Fig. 2), because off-target plastid data were not recovered for all taxa. While taxon sampling is limited (six species of *Senegalia* and one each of *Mariosousa*, *Parasenegalia* and *Pseudosenegalia*), it does represent a reasonable geo-taxonomic sampling of the group. The wider phylogeny showing the relationships of this senegalioid grade within the mimosoid clade (Fig. 2A, C) is presented in detail by Ringelberg et al. (2022). Here, we examine this new phylogenomic evidence presented by Ringelberg et al. (2022), re-visit the key morphological characters underpinning the two sections of *Senegalia* to see how they correspond to the new phylogeny and discuss the implications of these results for generic delimitation and taxonomy.

The two clades of *Senegalia* in this new phylogeny (Fig. 2B) are congruent with the two main *Senegalia* clades found by Bouchenak-Khelladi et al. (2010), Kyalangalilwa et al. (2013), Boatwright et al. (2015) and Terra et al. (2017) that had more comprehensive taxon sampling. In the nuclear phylogeny (Koenen et al. 2020; Ringelberg et al. 2022), the two well-supported clades correspond to: (1) a clade combining Senegaliasect.Senegalia, represented by *S.nigrescens* (Oliv.) P.J.H. Hurter and Senegaliasect.Monacanthea s.s. represented by *S.ataxacantha* (DC.) Kyal. & Boatwr. on the one hand (clade A in Terra et al. 2017); and (2) Senegaliasect.Monacanthea pro parte (p.p.) represented by *S.bahiensis* (Benth.) Seigler & Ebinger, *S.sakalava* (Drake) Boatwr., *S.borneensis* (I.C. Nielsen) Maslin, Seigler & Ebinger and *S.pentagona* (Schumach. & Thonn.) Kyal. & Boatwr. on the other hand (clade B in Terra et al. 2017). Furthermore, a third, moderately supported clade is shown that includes *Mariosousasericea* (M. Martens & Galeotii) Seigler & Ebinger, *Parasenegaliavisco* (Lorentz ex Griseb.) Seigler & Ebinger and *Pseudosenegaliafeddeana* (Harms) Seigler & Ebinger (Fig. 2B), interspersed between the two *Senegalia* clades. These three clades form a paraphyletic grade together with an accession that was included by Barneby and Grimes (1996) in *Albizialeonardii* Britton & Rose ex Barneby & J.W. Grimes (discussed below) and form successive sister groups to the remainder of the ingoid clade sensu Koenen et al. (2020). Thus, as shown by Koenen et al. (2020), the new analyses presented here (Ringelberg et al. 2022) show that, in the nuclear gene phylogeny, *Senegalia* is not monophyletic (Fig. 2B).

In marked contrast to the nuclear phylogeny, the two *Senegalia* sections are sister clades in the plastid phylogeny presented by Ringelberg et al. (2022), that supported the genus as monophyletic, based on plastid data alone (Fig. 2D). This cytonuclear conflict was also shown by Koenen et al. (2020) and probably explains why previous phylogenetic studies found *Senegalia* to be monophyletic because these studies either exclusively relied on plastid DNA sequences (Bouchenak-Khelladi et al. 2010; Kyalangalilwa et al. 2013; Boatwright et al. 2015) or primarily plastid genes in combination with limited taxon sampling outside *Senegalia* (Terra et al. 2017).

The analyses of Koenen et al. (2020) and Ringelberg et al. (2022) are based on DNA sequence data derived from 964 or 997 targeted nuclear genes, respectively, plus 72 plastid genes, datasets that are an order of magnitude larger than previous phylogenetic datasets. These large nuclear datasets provide robust support for the non-monophyly of *Senegalia*, with the two separate clades of *Senegalia* subtended by long, well-supported branches (Fig. 2B). However, notwithstanding the large number of genes underlying the analyses of Ringelberg et al. (2022), the backbone of this senegalioid grade is still characterised by extremely short branches (Fig. 2). Having data for many genes also means that conflict amongst individual gene trees can be examined and quantified, revealing high levels of gene tree conflict across the backbone of this senegalioid grade, but very high proportions of gene trees supporting each of the two *Senegalia* clades (Fig. 2B). These short branches across the senegalioid grade, combined with high levels of gene tree conflict across these nodes, plus the marked cytonuclear discordance suggest that alongside a rapid radiation characterised by incomplete lineage sorting, there may also be a history of reticulation, i.e. chloroplast capture or hybridisation. This is also evident from the unstable placements of *Albizialeonardii* and the *Parasenegalia*, *Pseudosenegalia* and *Mariosousa* clade across the different topologies presented by Ringelberg et al. (2022), where they swap places in the ASTRAL and amino acid RAxML phylogenies compared with the nucleotide RAxML phylogenies. This suggests that the branch subtending *Albizialeonardii* should be collapsed into a polytomy, as indicated by ASTRAL’s polytomy test (Fig. 2B) and the PhyloBayes consensus tree of Ringelberg et al. (2022), at least pending further phylogenomic evidence.

Given that the incongruence observed amongst the lineages of *Senegalia* and allies is likely caused by evolutionary processes, such as incomplete lineage sorting and introgression or chloroplast capture, this raises a number of fundamental questions about how to interpret these patterns: (1) how to define paraphyly vs. monophyly when there is pronounced cytonuclear discordance indicative of incomplete lineage sorting or reticulation and (2) is it justified to divide a genus into multiple segregate genera when the relationships amongst the constituent lineages are unresolved (i.e. form a potential hard polytomy)? With respect to the first question, we suggest that, given the propensity for plastid capture or introgression in plants (Larson et al. 2021; Rose et al. 2021) and the fact that the plastid genome is uniparentally inherited and represents a single largely non-recombining locus, relationships determined by a large set of nuclear genes provide a better basis for assessing monophyly and for delimiting taxa (Wei et al. 2021). With regard to the second question, lack of molecular resolution means that we should rely even more than usual on morphological diagnosability as the key criterion for delimiting lineages across a polytomy as separate genera. Thus, careful re-examination and evaluation of morphological variation across this grade of *Senegalia* and allies are needed to ascertain to what extent morphological data support the complex phylogenomic patterns revealed and provide the basis for an improved generic classification.

## Morphology and taxonomic implications

**Figure 3.**
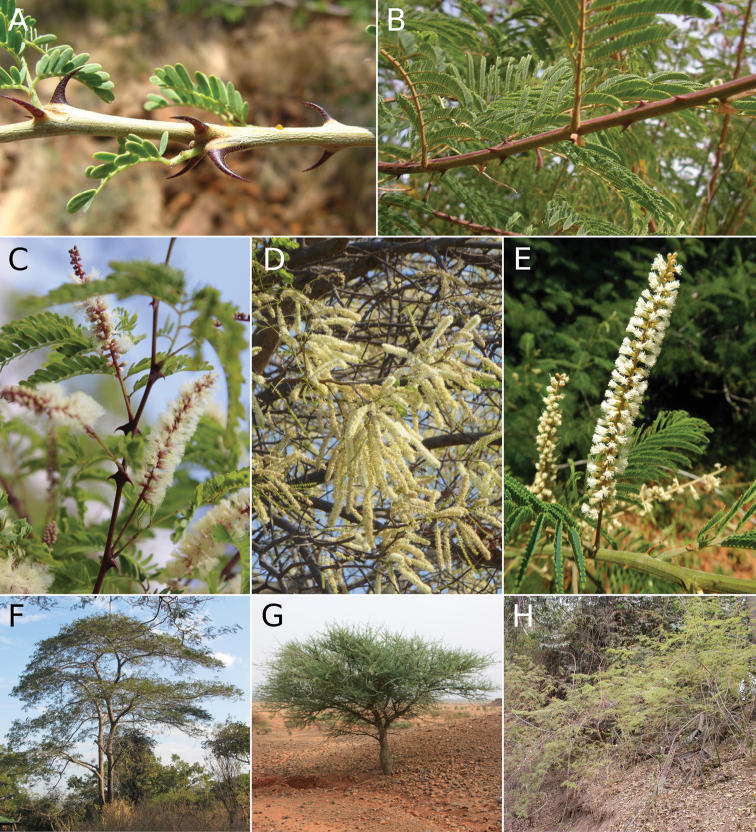
Key morphological features of the clade composed of Senegaliasect.Senegalia (**A, C, D, F, G**) and sect. Monacanthea s.s. (**B, E**, **H**). **A** prickles clustered at the nodes in *Senegaliasenegal* Britton **B** internodal prickles in *S.ataxacantha***C** paired prickles at the nodes and axillary, spicate inflorescences of *Senegaliagoetzei* (Harms) Kyal. & Boatwr. **D** spicate inflorescence in racemes of *Senegalianigrescens* (Oliv.) P.J.H. Hurter **E** spicate inflorescences of *S.ataxacantha***F** tree habit of *Senegaliapolyacantha* (Willd.) Seigler & Ebinger **G** treelet habit of *Senegalialaeta* (R. Br. ex Benth.) Seigler & Ebinger **H** lianescent shrub habit of *S.ataxacantha*. Photo credits: **A** Alex Dreyer **B** Sylvain Piry **C, D** Claude Boucher Chisale **E** Erik Koenen **F** Elke Faust **G** Marco Schmidt **H** Philippe Birnbaum **A–D, F–H** from African plants – A Photo Guide (www.africanplants.senckenberg.de) **E** from living collection Pretoria National Botanical Garden, South Africa.

**Figure 4.**
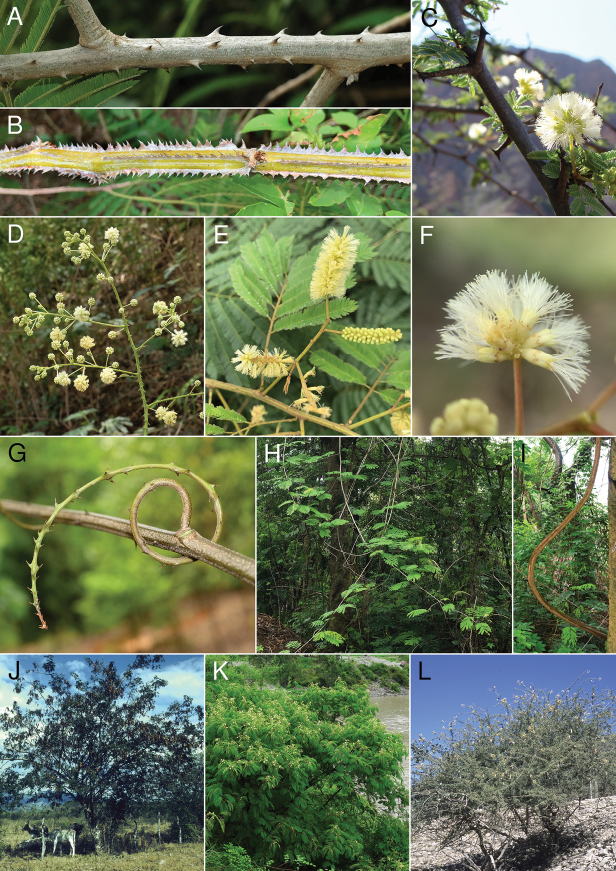
Key morphological features of the Senegaliasect.Monacanthea p.p. clade. **A** internodal prickles of Senegaliapennatasubsp.insuavis (Lace) Maslin, Seigler & Ebinger **B** internodal prickles of *Senegaliaserra* (Benth.) Seigler & Ebinger **C** paired forked spines at the nodes and capitate inflorescences of *S.gilliesii***D** paniculately compound inflorescence consisting of racemes of heads of *Senegaliaclandestina* Maslin, B.C. Ho, H. Sun & L. Bai **E** spikes in racemes of *Senegaliabonariensis* (Gillies) Seigler & Ebinger **F** capitate inflorescence of *Senegaliapolyphylla* (DC.) Britton & Rose **G** tendril with prickles of *Senegaliakunmingensis* (C. Chen & H. Sun) Maslin, B.C. Ho, H. Sun & L. Bai **H, I** liana habit of *Senegaliamegaladena* (Desv.) Maslin, Seigler & Ebinger **J** tree habit of *Senegaliapicachensis* Britton & Rose **K** shrub habit of *Senegaliateniana* (Harms) Maslin, Seigler & Ebinger **L** shrub habit of *S.gilliesii*. Photo credits: **A, D, G, H** Lin Bai B **F** Ítalo A.C. Coutinho **C** Guy Atchison **E** Vanessa Terra **I** Bruce Maslin **J, L** Colin Hughes **K** Hang Sun. Vouchers: **A***B.R. Maslin 11043***B***V. Terra & Í.A.C. Coutinho 701***C***G. Atchison 12*. **D***B.R. Maslin 11032***E***V. Terra & D.M.P. Pena 679***F***V. Terra & Í.A.C. Coutinho 683***G***L. Bai 2*, H, **I***B.R. Maslin 11040***J***C.E. Hughes 1416***K** unvouchered **L***C.E. Hughes 2306*.

The phylogenomic evidence, discussed here, shows that *Senegalia* is non-monophyletic and suggests that the two clades of *Senegalia* species (some of which are illustrated in Figs 3, 4) could potentially be treated as two (or possibly three) separate genera. Here, we evaluate the consistency of the morphological characters supporting the currently recognised sections, which largely correspond to the two recovered clades, except for the small *S.ataxacantha* group (sect. Monacanthea s.s.).

The disposition of cauline prickles (at or close to the leaf nodes in sect. Senegalia – Fig. 3A, C; vs. mostly internodal in sect. Monacanthea – Fig. 4A, B) and, to a lesser extent, inflorescence shape (usually spicate in sect. Senegalia – Fig. 3C, D, vs. globose, subglobose/oblongoid or sometimes spicate in sect. Monacanthea – Fig. 4C–F) and also plant growth form (always trees and shrubs in sect. Senegalia – Fig. 3F, G, vs. lianas, trees and shrubs in sect. Monacanthea – Fig. 4G–L) are the most informative characters for distinguishing the two sections. However, there are some exceptions (discussed below) and, furthermore, the practical utility of branchlet armature is somewhat diminished because cauline prickles are not infrequently absent from individual plants or herbarium specimens in species where they are otherwise known to exist. Nevertheless, what is clear is that prickles are present in all species of *Senegalia*, while they are absent in the three allied genera in the New World, namely *Mariosousa*, *Parasenegalia* and *Pseudosenegalia* (fide Miller et al. 2017).

**Figure 5.**
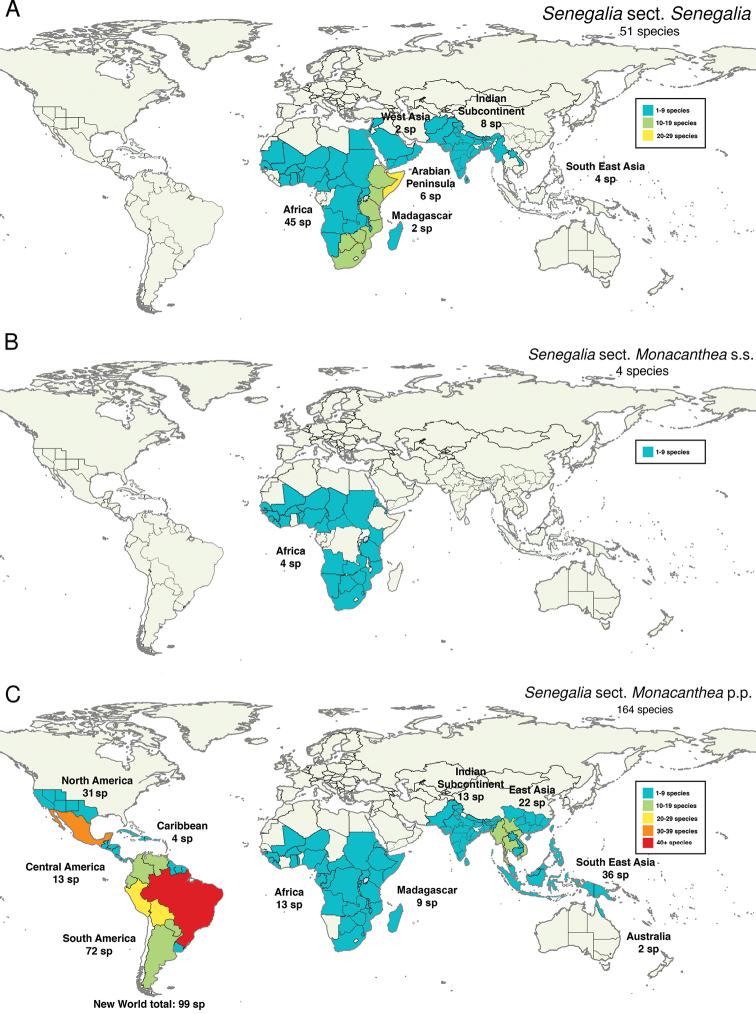
Distribution of the main groups of *Senegalia***A**Senegaliasect.Senegalia. **B**Senegaliasect.Monacanthea s.s. **C**Senegaliasect.Monacanthea p.p. Species numbers derived from Maslin and Wilson (2021) at WorldWideWattle website (http://worldwidewattle.com).

The Afro-Asian sect. Senegalia contains 51 species distributed in Africa, the Arabian Peninsula, West Asia and the Indian Subcontinent to Myanmar and Laos in Southeast Asia, with the greatest diversity of species in Somalia in the Horn of Africa (Fig. 5A, Table 1). Morphologically, the species of this section appear to be relatively invariable. Importantly, most possess between one and three prickles at or near the leaf nodes (Fig. 3A, C) and invariably lack internodal prickles. The only exceptions known to us are two African species documented by Ross (1979) in his conspectus of what was then African *Acacia*. First, *Senegaliapseudonigrescens* (Brenan & J.H. Ross) Kyal. & Boatwr. was described as unarmed, but this species was known only from the type, which could be anomalous and examination of additional material is needed to verify this observation. Second, *Senegaliacaffra* (Thubb.) P.H. Hurter & Mabb. was described as rarely having a few scattered prickles on the internodes, in addition to the pair located at the nodes. Flowers in 95% of species of sect. Senegalia are aggregated in spikes (containing sessile flowers) or occasionally spiciform racemes (i.e. spikes with pedicellate flowers), while globose or sometimes oblongoid heads occur in only two African taxa, *S.densispina* (Thulin) Kyal. & Boatwr. and S.melliferasubsp.detinens (Burch.) Kyal. & Boatwr. In the majority of species of sect. Senegalia, the inflorescences are axillary, but occasionally they are arranged in racemes or panicles, e.g. *S.burkei* (Benth.) Kyal. & Boatwr. and *S.caffra* from Africa. Unlike many species of sect. Monacanthea, lianas are never found amongst the species of sect. Senegalia (which are either shrubs or trees, Fig. 3F, G).

**Table 1.** Major groups of the senegalioid grade showing species numbers and regional distribution derived from Maslin and Wilson (2021) at WorldWideWattle website (http://worldwidewattle.com). Note: As used here, North America includes Mexico and the U.S.A. and Asia includes New Guinea.

Name	Total species number	Distribution (species number)
*Senegalia*	219	**Pantropical**
Sect. Senegalia	51	**Old World only. African region**: Africa (45 spp.), Madagascar (2 spp.), Arabian Peninsula (6 spp.); **Asian region**: West Asia (2 spp.), Indian subcontinent (8 spp.), Southeast Asia (4 spp.)
Sect. Monacanthea p.p.	164	**Pantropical: New World**: Caribbean (4 spp.), Central America (13 spp.), North America (31 spp.), South America (72 spp.); **African region**: Africa (13 spp.), Madagascar (9 spp.); **Asian region**: Indian subcontinent (13 spp.), East Asia (22 spp.), Southeast Asia (36 spp.); **Australia** (2 spp.)
Sect. Monacanthea s.s	4	**Africa** (4 spp.)
*Mariosousa*	14	**New World**: Central America (3 spp.), North America (13 sp.)
*Parasenegalia*	11	**New World**: Caribbean (3 spp.), Central America (1 sp.), South America (7 spp.)
*Pseudosenegalia*	2	**New World**: South America – endemic to Bolivia (2 spp.)

The much larger pantropical sect. Monacanthea p.p. contains 164 species (excluding the four species of sect. Monacanthea s.s., see below) distributed in the Americas (especially Brazil with 63 species), Africa, Asia and Australia (Fig. 5C, Table 1). Section Monacanthea p.p. is not only more speciose and geographically widespread than sect. Senegalia, but also morphologically more variable, especially in the New World. The best morphological feature for distinguishing sect. Monacanthea p.p. from sect. Senegalia appears to be cauline prickle distribution, mostly internodal in sect. Monacanthea p.p. (Fig. 4A, B) and nodal in sect. Senegalia (Fig. 3A, C). However, in the New World (where sect. Senegalia is not known to occur), there is some variation in armature that appears to reduce the discriminating value of this character in that region and which warrants further investigation. Of the 99 species New World species of Senegaliasect.Monacanthea p.p. there are 28 species that possess nodal prickles, at least some of the time. For example, it is not uncommon to find specimens, especially amongst species in the informal *S.berlandieri* species-group and *S.monacantha* (Willd.) Seigler & Ebinger from the informal *S.monacantha* species-group, which have 1–3 prickles associated with some or most nodes, in addition to those on the internodes. Furthermore, the following three species have one or two prickles associated with some nodes, but none on the internodes, namely, *S.emilioana* (Fortunato & Ciald.) Seigler & Ebinger, *S.gilliesii* (Steud.) Seigler & Ebinger (Fig. 4C) and *S.subsessilis* Britton & Rose from the informal *S.greggii* (A. Gray) Britton & Rose species-group.

Inflorescence shape (i.e. globose or occasionally subglobose/oblongoid heads vs. cylindrical spikes) is a useful character for distinguishing sect. Monacanthea p.p. from sect. Senegalia, but again, some New World species differ somewhat from those elsewhere. In the Afro-Asian region where the two sections co-occur, 92% of sect. Monacanthea p.p. species possess globose/oblongoid heads (Fig. 4D), contrasting with sect. Senegalia where almost all species possess cylindrical spikes (see above). Of the five sect. Monacanthea p.p. species from this region that possess spikes, three are from Madagascar [*S.hildebrandtii* (Vatke) Boatwr., *S.menabeensis* (Villiers & Du Puy) Boatwr. and *S.pervillei* (Benth.) Boatwr.] and two are from Southeast and East Asia [*S.donnaiensis* (Gagnep.) Maslin et al. and *S.yunnanensis* (Franch.) Maslin et al., respectively]. In the New World (where sect. Senegalia is not known to occur), there is a stronger bias towards spicate inflorescences within sect. Monacanthea p.p. where 26 species (representing 27% of the species from the region) possess spikes (Fig. 4E), while 70 species (71%) possess globose or sometimes subglobose heads (Fig. 4C, F). It is noted that several other mimosoid genera that are known to be monophyletic are polymorphic with regards to these inflorescence shapes (e.g. *Inga* Mill., *Zygia* P. Browne., *Mimosa* L., *Acacia*, *Parkia* R. Br.). Globally, a higher percentage of sect. Monacanthea p.p. species also has inflorescences arranged in terminal racemes or panicles than those with inflorescences arising from the axils of leaves, contrasting with sect. Senegalia where most species have axillary inflorescences. In sect. Monacanthea p.p., lianas are relatively common (Fig. 4G–I), whereas in sect. Senegalia, they never occur. However, sect. Monacanthea p.p. also includes trees and shrubs (Fig. 4J–L), especially in the New World.

*Senegaliaataxacantha* and its three relatives, *S.macrostachya* (Rchb. ex DC.) Kyal. & Boatwr., *S.chariessa* (Milne-Redh.) Kyal. & Boatwr. and *S.eriocarpa* (Brenan) Kyal. & Boatwr. have consistently been placed in the same clade as sect. Senegalia in all phylogenetic studies that included one or other of these species (i.e. Bouchenak-Khelladi et al. 2010; Kyalangalilwa et al. 2013; Boatwright et al. 2015; Terra et al. 2017; Koenen et al. 2020; Ringelberg et al. 2022), as well as in the morphological cladistic study of Chappill and Maslin (1995). This group is referred to herein as sect. Monacanthea s.s., because it includes the type of the section (*S.ataxacantha*), while the remainder of the section is referred to as sect. Monacanthea p.p. However, unlike species of sect. Senegalia that have prickles located at or near the leaf nodes, these four African species (Fig. 5C) possess internodal cauline prickles (Fig. 3B; although sometimes in *S.chariessa*, a few prickles may also be grouped irregularly in pairs near the nodes), similar to the majority of species of sect. Monacanthea p.p. Indeed, Vassal (1972) united the species with scattered internodal prickles in this section and treated *Acaciaataxacantha* (≡ *Senegaliaataxacantha*) as the type of the section (which has potential nomenclatural implications, depending upon how *Senegalia* is classified in the future). These four species have flowers arranged in spikes (Fig. 3E), a character almost ubiquitous for sect. Senegalia, but rare in other Afro-Asian species of sect. Monacanthea p.p. Finally, these species have a lianescent or scrambling shrubby habit (Fig. 3H) that is not seen in sect. Senegalia, while a liana or lianescent habit is relatively common in species of sect. Monacanthea p.p. (as discussed above). These intermediate characteristics of sect. Monacanthea s.s. are in line with their phylogenetic placement as either sister to sect. Senegalia or sister to the rest of sect. Senegalia minus *S.catechu* (L.f.) P.J.H. Hurter & Mabb. (Kyalangalilwa et al. 2013; Boatwright et al. 2015; Terra et al. 2017). Given the phylogenetic position and morphological characteristics of sect. Monacanthea s.s., this group could either be regarded as a distinct genus or remain classified within the genus Senegaliaalongsidesect.Senegalia; these matters require further investigation. It also appears that the large majority of Senegalia species currently placed in sect. Monacanthea p.p. will need to be reclassified as a separate genus, pending further phylogenomic studies with increased taxon sampling (as outlined below). The name *Manganaroa*Spegazzini (1923) is available for this genus, but its type species, the South American *S.monacantha*, while placed in sect. Monacanthea p.p., has not so far been included in any phylogenetic study. In any case, it is clear that this discordant combination of internodal prickles and spicate inflorescences undermines the potential of these two characters to straightforwardly diagnose the two major clades of *Senegalia* (Figs 3, 4).

***Albizialeonardii***

The phylogenetic placement of *Albizialeonardii* amongst *Senegalia* and allies (Fig. 2B) raises questions about its identity and the identity of the material used in the Ringelberg et al. (2022) analysis under this name. This species was described by Barneby and Grimes (1996) who acknowledged that, in the absence of having adequate flowering material, even the generic placement of their new entity was uncertain. Our examination of online images of *Leonard 7490*, the holotype of *A.leonardii* at the US Herbarium, a fragment of the isotype at NY and a paratype (*Zanoni 34986*, NY, material that was used in the present phylogenomic studies), reveals that these collections show a suite of vegetative characters that are consistent with those of *Parasenegaliavogeliana* (Steud.) Seigler & Ebinger: there are no prickles on the branches; the petiolar nectary has the same position and shape; similar leaflet shape, size, apex, base and veins; number of pinnae pairs (about 4) and number of leaflets per pinna (about 10). This is in line with the determination of the US holotype as *Lysilomavogelianum* (Steud.) Stehlé, which is a homotypic synonym of *Parasenegaliavogeliana* (Seigler et al. 2017) and with the close geographical proximity of the type localities of *A.leonardii* and *P.vogeliana* in Haiti. Despite the lack of flowers on any of the material of *A.leonardii* referred to above, the combined morphological similarities coinciding with geographical proximity of type localities and the phylogenetic placement of *A.leonardii* (Fig. 2B), support treatment of *A.leonardii* as a synonym of *P.vogeliana*. This result implies that *Parasenegalia* is also potentially non-monophyletic, adding further to the difficulties of delimiting genera across *Senegalia* and allies. Given such a significant consequence, it is recommended that sequencing of additional *Parasenegalia* species, preferably using the *Mimobaits* gene set of Koenen et al. (2020), is needed to further assess the potential non-monophyly of *Parasenegalia*. This is especially relevant, given the lack of support for the genus and the extremely short branch subtending *Parasenegaliavisco* (which is also morphologically somewhat anomalous) in the phylogenetic analysis of Miller et al. (2017).

***Parasenegaliavogeliana* (Steud.) Seigler & Ebinger in Seigler et al., Novon 25(2): 197–199, fig. 9. 2017.**

*Acaciaambigua* Vogel, Linnaea 10: 600–601. 1836, nom. illeg., non *Acaciaambigua* Hoffmanns., Zweit. & Dritt. Nacht. Verz. Pfl.-Kult., [3^rd^ addendum] 15. 1826. Type: B†.

*Acaciavogeliana* Steud., Nomencl. Bot. [Steudel], ed. 2,1: 9. [Aug.] 1840, replacement name for *Acaciaambigua* Vogel, Type: Based on *Acaciaambigua* Vogel.

*Senegaliavogeliana* (Steud.) Britton & Rose, N. Amer. Fl. 23(2): 116. [25 Sep.] 1928. Type: Based on *Acaciaambigua* Vogel.

*Lysilomavogelianum* (Steud.) Stehle, Bull. Mus. Natl. Hist. Nat., sér. 2, 18(2): 193–194. 1946. Type: Based on *Acaciaambigua* Vogel.

**Type material.** Haiti. Santo Domingo: Plaine prés de Port-au-Prince, Ramuli partem cl. Ehrenberg misit tantum summam; 1828–1839, *C.A. Ehrenberg 274* (lectotype, designated by Seigler et al. 2006, pg 79: HAL [HAL0040798] [fr.], HAL photo at K; isolectotypes, B fragm. at US [US000000564], B photo at K; NY [NY00001533].

=*Albizialeonardii* Britton & Rose ex Barneby & J. W. Grimes. syn. nov. 1996. Silk Tree, Guanacaste, Monkey’s Earring, Memoirs of the New York Botanical Garden, Volume 74, Part 1, p 216.

**Type materials.** Haiti. Dept. du Nord; Habilitation Baille n of Atalaye Plantation, S. Michel de l’Atalaye, in dry thicket; 350 m alt.; 26 Nov 1925. *E. C. Leonard 7490*. Holotype: US; isotype (fragment of holotype) + photo of holotype, NY. Paratype: Haiti. Dept. Artibonite: Dubedou (de Gonaives), 20 km al N. de Gonaives en la carretera a Port-de Paix, zona arida; 130 m alt.; 8 Jun 1985; [young bud]; *T. Zanoni et al. 34986* (JBSD, NY).

## Conclusions

All phylogenetic studies have shown that *Senegalia* comprises two, robustly supported clades, which largely correspond to sect. Senegalia and sect. Monacanthea, but with the exception of the *S.ataxacantha* group (sect. Monacanthea s.s.) which aligns with sect. Senegalia. The recent phylogenomic analyses, discussed here, show that these two clades are not sister groups and that *Senegalia* is non-monophyletic supporting the possible recognition of these clades as separate genera, based on nuclear data. We anticipate that *Senegalia* will indeed need to be re-classified to reflect this non-monophyly. However, it is also clear that the key morphological traits distinguishing these two clades, namely, armature and, to a lesser extent, inflorescence shape, are not totally consistent across the majority of species within these clades. The most notable inconsistency presently known is the small African *S.ataxacantha* group that is morphologically discordant with the phylogenetic evidence and whether this group is most appropriately treated as a separate genus or retained within the genus *Senegalia* remains to be decided. Given that only about 75 of the total 219 species of *Senegalia* have so far been included in phylogenies (with only six in the recent phylogenomic studies) and that a number of morphologically anomalous species have not yet been sampled for molecular data, it is clear that splitting *Senegalia* at this point would be premature, especially given the nomenclatural repercussions involving name changes for 164 species on four continents. More species of the two clades of *Senegalia* and the allied genera *Mariosousa*, *Parasenegalia* and *Pseudosenegalia* need to be sequenced and an in-depth investigation of possible reticulate patterns, including with the use of phylogenetic network analysis, should be carried out before any decisions regarding formal taxonomic rearrangements are made. Consequently, below we provide a list of critical taxa for future sequencing, ideally to be carried out using the *Mimobaits* nuclear gene set of Koenen et al. (2020).

## Critical taxa for inclusion in future phylogenomic studies

The following species are suggested for inclusion in future phylogenomic studies to achieve taxon sampling that is geographically, morphologically and taxonomically representative of *Senegalia*. In addition, denser sampling of taxa across the allied genera *Mariosousa*, *Parasenegalia* and *Pseudosenegalia* is needed.

1. *Mariosousa* species: *M.centralis* (Britton & Rose) Seigler & Ebinger, *M.coulteri* (Benth.) Seigler & Ebinger.
2. *Parasenegalia* species: *P.miersii* (Benth.) Seigler & Ebinger, *P.muricata* (L.) Seigler & Ebinger, *P.rurrenabaqueana* (Rusby) Seigler & Ebinger, *P.vogeliana* (Steud.) Seigler & Ebinger.
3. *Pseudosenegaliariograndensis* (Atahuachi & L. Rico) Seigler & Ebinger.
4. *Senegalia* species:

- Africa (sect. *Monacanthea* p.p. species with globose or oblongoid heads): *S.brevispica* subsp. *brevispica* (Harms) Seigler & Ebinger, *S.schweinfurthii* (Brenan & Exell) Seigler & Ebinger (either variety).
- Africa (sect. *Monacanthea* s.s.): **Ataxacantha species-group**: *S.eriocarpa*, *S.chariessa*, *S.macrostachya*.
- Africa (sect. *Senegalia*): *S.burkei* or *S.caffra* (inflorescences racemes or panicles), *S.densispina* (heads globose), *S.erubescens* (Welw. ex Oliv.) Kyal. & Boatwr., *S.laeta* (R. Br. ex Benth.) Seigler & Ebinger or *S.rovumae* (Oliv.) Kyal. & Boatwr. (inflorescence, a spiciform raceme), *S.senegal* (L.) Britton (any variety), *S.mellifera* (Vahl) Seigler & Ebinger.
- Americas (sect. *Monacanthea* p.p.): **Amazonica species-group**: *S.amazonica* (Benth.) Seigler & Ebinger, *S.serra* (Benth.) Seigler & Ebinger; **Berlandieri species-group**: *S.berlandieri* (Benth.) Britton & Rose, *S.bonariensis* (Gillies ex Hook. & Arn.) Seigler & Ebinger, *S.gaumeri* (S.F. Blake) Britton & Rose or *S.langsdorffii* (Benth.) Seigler & Ebinger, *S.kelloggiana* (A.M. Carter & Rudd) C.E. Glass & Seigler, *S.paganuccii* Seigler, Ebinger & P.G. Ribeiro, *S.picachensis* (Brandegee) Britton & Rose or *S.interior* Britton & Rose; **Greggii species-group**: *S.emilioana*, *S.gilliesii*, *S.greggii*, *S.occidentalis* (Rose) Britton & Rose, *S.subsessilis*; **Martiusiana species-group**: *S.martiusiana* (Steud.) Seigler & Ebinger; **Monacantha species-group**: *S.monacantha*; **Pedicellata species-group**: *S.pedicellata* (Benth.) Seigler & Ebinger; **Polyphylla species-group**: *S.polyphylla* (DC.) Britton & Rose; **Riparia species-group**: *S.riparia* (Kunth) Britton & Rose; **Tamarindifolia species-group**: *S.tamarindifolia* (L.) Britton & Rose; **Tenuifolia species-group**: S. *mirandae* (L. Rico) Seigler & Ebinger, *S.tenuifolia* (L.) Britton & Rose. **Unplaced in any group**: *S.kallunkiae* (J.W. Grimes & Barneby) Seigler & Ebinger, *S.piptadenioides* (G.P. Lewis) Seigler & Ebinger, *S.ricoae* (Bocage & Miotto) L.P. Queiroz , *S.weberbaueri* (Harms) Seigler & Ebinger.
- Asia (sect. *Monacanthea* p.p.): **Caesia species-group**: *S.caesia* (L.) Maslin, Seigler & Ebinger; **Hainanensis species-group**: *S.hainanensis* (Hayata) H. Sun and/or *S.pluricapitata* (Steud. ex Benth.) Maslin, Seigler & Ebinger; **Pennata species-group**: *S.kerrii* (I.C. Nielsen) Maslin, B.C. Ho, H. Sun & L. Bai, *S.pennata* (L.) Maslin (either or both subspecies) or *S.megaladena* (Desv.) Maslin, Seigler & Ebinger (either subspecies); **Rugata species-group**: *S.rugata* (Lam.) Britton & Rose; **Teniana species-group**: *S.kunmingensis* (C. Chen & H. Sun) Maslin, B.C.Ho, H.Sun & L.Bai or *S.prominens* Maslin, B.C. Ho, H. Sun & L. Bai, *S.yunnanensis*; **Unplaced in any group**: *S.donnaiensis*, *S.kostermansii* (I.C. Nielsen) Maslin, Seigler & Ebinger, *S.kekapur* (I.C. Nielsen) Maslin, Seigler & Ebinger, *S.thailandica* (I.C. Nielsen) Maslin, Seigler & Ebinger.
- Asia: (sect. *Senegalia*) : *S.catechu*, *S.modesta* (Wall.) P.J.H. Hurter.
- Madagascar (sect. *Monacanthea* p.p. species with spikes): *S.menabeensis*.
